# Entire Encapsulation of Thymopentin by Extended Biphen[3]arene Carboxylate for Improving Plasma Stability

**DOI:** 10.3390/molecules30020314

**Published:** 2025-01-15

**Authors:** Keming Ren, Junyi Chen, Chunju Li

**Affiliations:** 1 Second Clinical Medical College, Heilongjiang University of Chinese Medicine, Harbin 150006, China; rendonggua999@gmail.com; 2 Academy of Interdisciplinary Studies on Intelligent Molecules, College of Chemistry, Tianjin Normal University, Tianjin 300387, China; cjli@shu.edu.cn

**Keywords:** peptide drug, macrocyclic host, molecular recognition, plasma stability

## Abstract

Peptide-based therapy is appealing in modern medicine owing to its high activity and excellent biocompatibility. Poor stability, leading to unacceptable bioavailability, severely constrains its clinical application. Here, we proposed a general supramolecular approach for improving the plasma resistance of a commercially available peptide agent, thymopentin. The ^1^H NMR results indicated that the large-sized extended biphen[3]arene carboxylate (ExBP3C) can entirely encapsulate this peptide at its main chain with a binding stoichiometry of 1:1 and *K*_a_ value of (1.87 ± 0.15) × 10^5^ M^−1^, which varied radically from recognizing specific amino acid residues by carboxylatopillar[5]arene (CP5A). Notably, host–guest complexation by ExBP3C could maintain 24.85% of the original thymopentin amount for 60 min in the presence of rat plasma, whereas free thymopentin, or co-dosed with CP5A and cucurbit[7]uril, underwent rapid degradation and became undetectable within just 30 min. In addition, cytotoxicity and hemolysis assays preliminary demonstrated that the employment of ExBP3C as a supplementary material was relatively nontoxic at a cellular level.

## 1. Introduction

Peptides affect many important physiological and biochemical functions in living organisms [1,2]. They can serve as neurotransmitters, neuroregulatory factors, and hormones participating in receptor-mediated signal transduction [3,4,5]. Peptides are also able to interact with receptors to influence intercellular information exchange and are involved in lots of biochemical processes [6,7]. With a deeper understanding of the action mode of biologically active peptides, researchers have produced a growing interest in its application in pharmacology and medicine. In view of their high activity and good biocompatibility, currently, some pre-eminent peptide agents have been placed into clinical practice or the preclinical stage, covering an extended range of therapeutic fields, e.g., cancer, diabetes, immune disease, cerebro-cardiovascular disease [8,9,10,11,12]. However, poor stability, leading to unacceptable bioavailability, severely constrains its clinical application [13,14]. To alleviate such bottlenecks, many methods have been developed to improve their metabolic stability, for instance, substitution by non-natural amino acids [15], preparation of cyclopeptide or staple peptides [16,17], and employment of supplementary materials [18,19].

Alternatively, the recognition of bioactive substances within macrocycle cavities has been demonstrated to be a feasible approach to regulating their functions [20,21]. While strictly constrained to cavity dimensions, traditional macrocycles are commonly feasible for complex small- or medium-sized guests [22,23,24]. Important biomacromolecules, like peptides, can only recognize specific amino acids from side chains [25,26,27,28,29]. One thing should be mentioned: the enzyme digestion sites of peptides are commonly located at the amide linkage to the main chain, and the above mode of recognizing residues cannot effectively exert protection outcomes [30,31]. This work is aimed at describing a general supramolecular approach to improving the plasma resistance of peptide agents via entire encapsulation at its main chain by large-sized macrocycles.

**Scheme 1:**
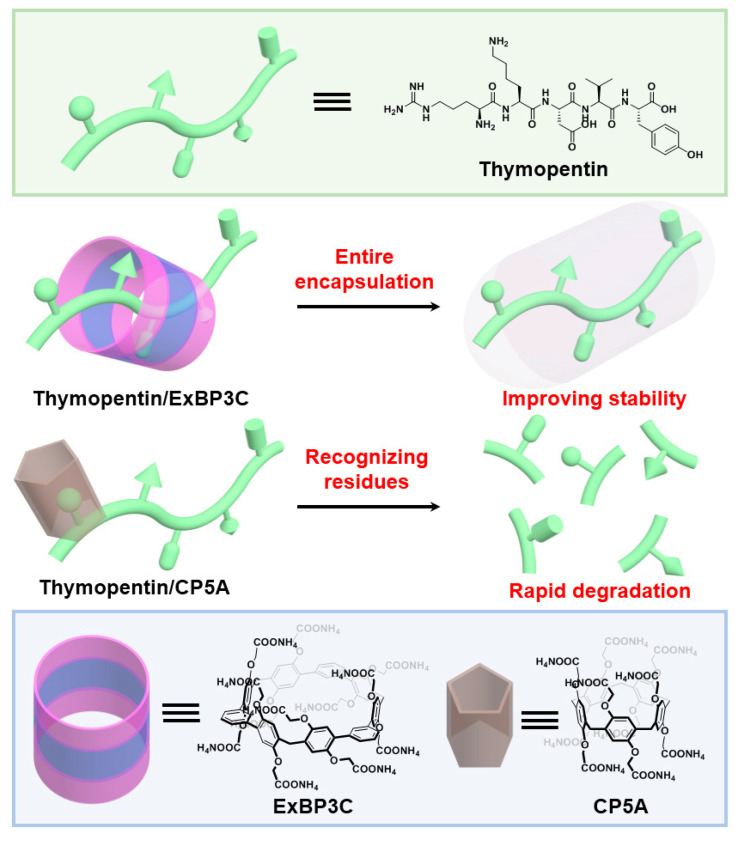
Chemical structures of thymopentin, extended biphen[3]arene carboxylate (ExBP3C), and carboxylatopillar[5]arene (CP5A), and schematic illustration of the impact of different recognition modes by macrocycles on plasma stability of thymopentin.

As a proof of concept, thymopentin, a clinical agent for the treatment of immunodeficiency diseases, such as cancers, acquired immunodeficiency syndrome, hepatitis B virus infection, and rheumatoid arthritis, served as a model peptide [32,33]. Extended biphen[3]arene carboxylate (ExBP3C), customized by a modular synthetic strategy, was chosen as the macrocyclic host due to its large-sized cavity and distinctive recognition property [34]. Such a macrocycle could entirely encapsulate thymopentin at the main chain to improve its metabolic stability in the presence of rat plasma. In contrast, a popular macrocycle, carboxylatopillar[5]arene (CP5A), has been demonstrated to be unable to effectively protect thymopentin from degradation via recognizing specific amino acid residues under the same experimental conditions (Scheme 1). Such large-sized macrocycles are posed for further development as versatile tools to regulate the functions of biological macromolecules via host–guest interactions.

## 2. Results and Discussion

**Figure 1:**
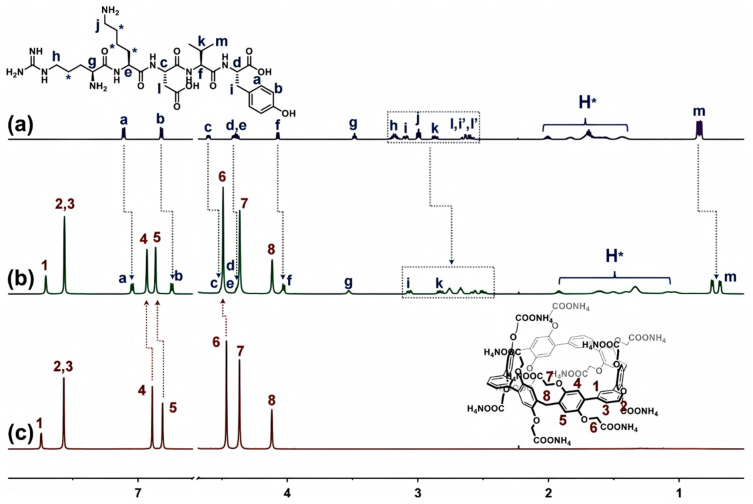
^1^H NMR spectra (600 MHz, D_2_O) of (**a**) thymopentin (5.0 mM), (**b**) thymopentin (5.0 mM) with the addition of ExBP3C (5.0 mM), and (**c**) ExBP3C (5.0 mM).

The designed predicate underlying the present approach for improving the plasma stability of peptides is that the large-sized ExBP3C, which displayed a geometric configuration of a regular hexagon with a side length of 7.2 Å, would recognize thymopentin at the main chain via the entire encapsulation mode. We thus performed ^1^H NMR spectroscopy to verify this specific complexation behavior. Figure 1 shows the spectra of thymopentin in D_2_O in the absence and presence of an equivalent of ExBP3C. First, the proton signals of tertiary carbon on the main chain of thymopentin (H_c_, H_d_, H_e_, and H_f_) underwent a discernible upfield shift and experienced broadening when mixed with ExBP3C. Next, all proton peaks of flexible sidechains exhibited various degrees of upfield shifts and broadening effects compared with free thymopentin. More importantly, the well-defined proton signals of tyrosine (Tyr) and valine (Val), which commonly cannot be recognized by traditional macrocycles, i.e., carboxylatopillar[5]arene (CP5A), shifted upfield (∆*δ* = 0.07, 0.08, and 0.11 ppm for H_a_, H_b_, and H_m_). Meanwhile, the peaks for ExBP3C shifted downfield due to the complexation-induced deshielding effect. Taken together, the above changes of ^1^H NMR spectra led us to suggest that the capacious ExBP3C might be fitted to encapsulate thymopentin entirely at the main chain. The possible interaction sites of thymopentin/ExBP3C included a hydrophobic area between the macrocyclic cavity and alkyl chain and a salt bridge between the carboxylate moiety and the positively charged terminal group.

**Figure 2:**
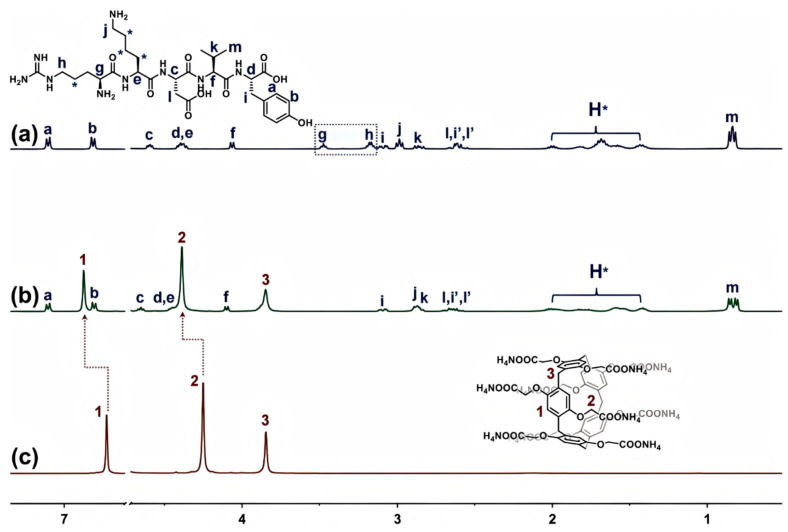
^1^H NMR spectra (400 MHz, D_2_O) of (**a**) thymopentin (5.0 mM), (**b**) thymopentin (5.0 mM) with the addition of CP5A (5.0 mM), and (**c**) CP5A (5.0 mM).

Previous research has reported that smaller-sized macrocycles bond to peptides by recognizing specific amino acids from side chains. Among them, CP5A, with an internal cavity size of ~4.7 Å, was demonstrated to selectively interact with arginine (Arg) or lysine (Lys) [26], and the exact sequence of thymopentin contains these two amino acids. Hence, the recognition mode of thymopentin with CP5A was further studied for comparison. As shown in Figure 2, upon the addition of CP5A, the proton peaks of the flexible sidechains of Arg and Lys displayed upfield shifts and broadening effects, and, particularly, signals for H_h_ and H_g_ of Arg disappeared. It was reasonable that the association constant (*K*_a_) of Arg was larger than that of Lys, and adjacent negatively charged aspartic acid (Asp) could produce a strong coulombic repulsion against multiple carboxylate moieties of CP5A. Another possible explanation for the preferential Arg encapsulation demonstrated in Figure 2 could be the aromatic-π character of the guanidinium group in Arg. This is in contrast to Lys, which is a primary amine and does not have the additional pi–pi interactions with the aromatic rings of the CP5A. Notably, the H_a_,_b_ of Tyr and H_m_ of Val were basically unchanged, revealing that CP5A interacted with thymopentin mainly via recognizing the residue of Arg.

**Figure 3:**
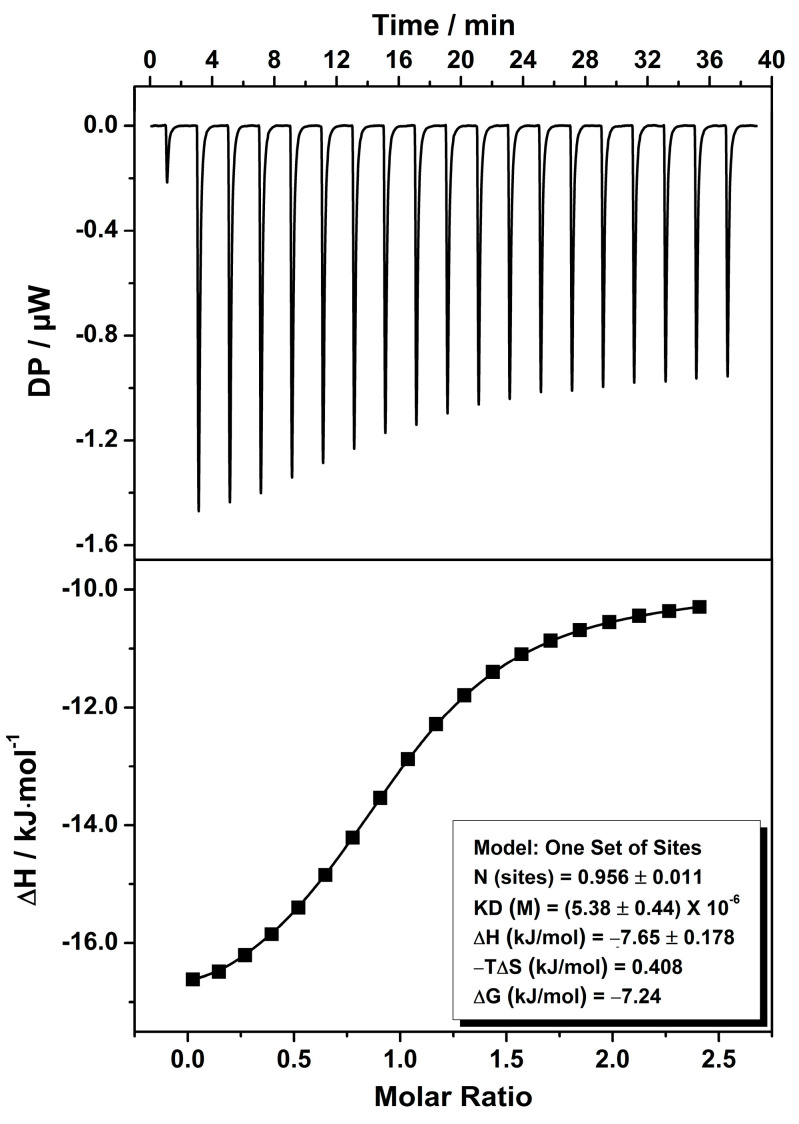
ITC isotherm for the titration of thymopentin/ExBP3C in aqueous solution at room temperature (**top**: raw data; **bottom**: net reaction heat obtained from integration of calorimetric traces).

An isothermal microcalorimetric titration (ITC) experiment was further carried out in deionized water at 25 °C on a PEAQ-ITC to quantitatively assess the binding affinity and related thermodynamic parameters. A 300 μL solution of ExBP3C was placed in the sample cell at a concentration of 40 μM, and 70 μL of a solution of thymopentin (500 μM) was placed in the injection syringe. The titration was conducted by titration of 18 drops of thymopentin to the ExBP3C solution, and all solutions were degassed prior to titration. The experimental results revealed that the recognition process was predominantly governed by enthalpic gains (∆*H* < 0) and unfavorable entropic losses (∆*S* < 0), yielding a *K*_a_ value of (1.87 ± 0.15) × 10^5^ M^−1^. It should be noted that the binding stoichiometry between ExBP3C and thymopentin was determined to be 0.956 ± 0.011, which was very close to 1:1 and consistent with the above entire encapsulation mode (Figure 3). A larger interaction area of thymopentin/ExBP3C would facilitate the production of a stronger binding affinity.

**Figure 4:**
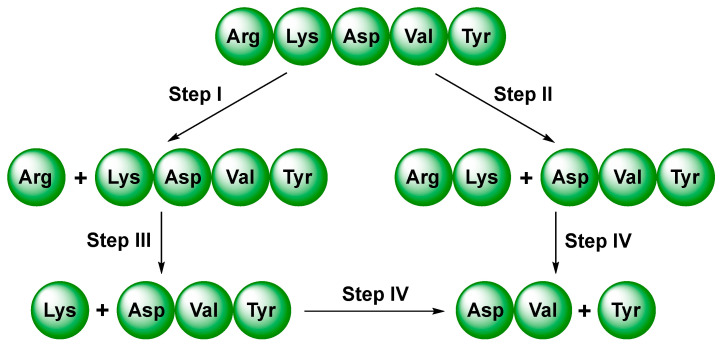
Possible scheme for degradation of thymopentin in plasma.

Previously reported studies have shown that thymopentin was susceptible to enzymes that degraded the peptide bonds adjacent to Arg and Lys, and the cleavage of the Val–Tyr bond proceeded more slowly. As shown in Figure 4, degradation steps I, II, and III appeared to be much more rapid than step IV [35]. Traditional macrocycles commonly interact with peptides via engulfing at the side chain of specific amino acids rather than amide linkage at the main chain. The enzyme digestion sites are located just at the amide bond, and thus, complexation by traditional macrocycles frequently leads to a poor effect on the protection potency of peptides. In view of the above favorable findings, we have reason to believe that entire encapsulation by ExBP3C could effectively improve the tolerance of thymopentin against enzymes.

**Figure 5:**
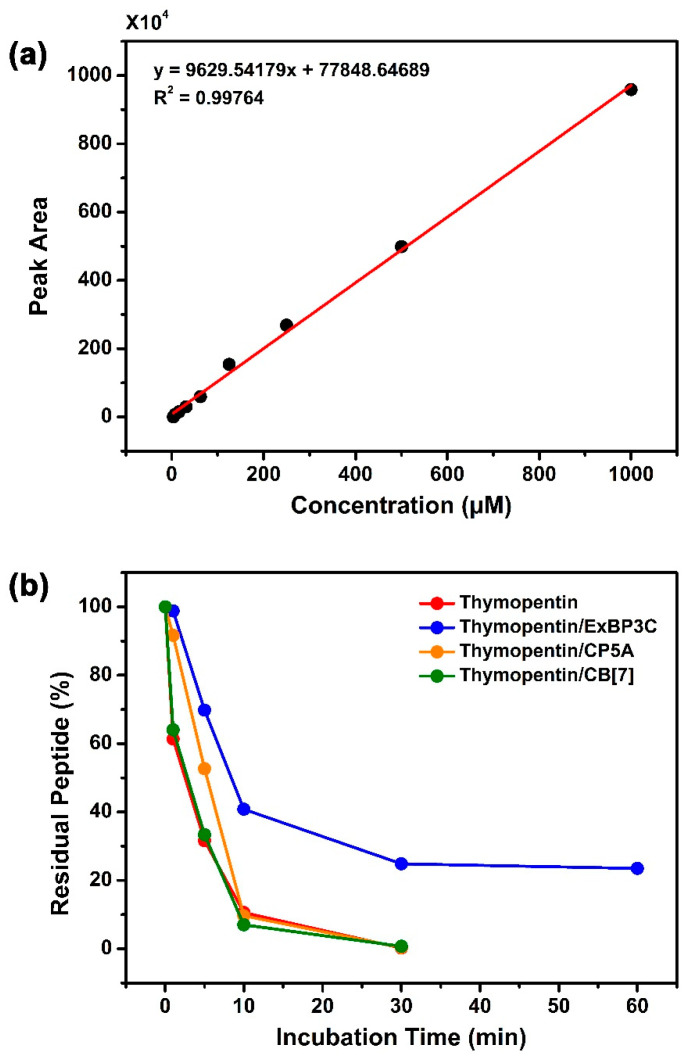
(**a**) Calibration curve obtained by HPLC and used to calculate thymopentin concentration in plasma stability. (**b**) Residual percentage of thymopentin (1.0 mM) in rat plasma in the absence and presence of ExBP3C (2.0 mM), CP5A (2.0 mM), and CB[7] (2.0 mM) as determined by HPLC.

To test the above hypothesis, we compared the plasma stability of free thymopentin, a host–guest mixture of thymopentin/ExBP3C, thymopentin/CP5A, and thymopentin/cucurbit[7]uril (CB[7]) using high-performance liquid chromatography (HPLC). According to a previously reported method [36], the above samples (all containing 1.00 mM of thymopentin) were dissolved in deionized water and then incubated with an equal amount of rat plasma at 37 °C. At predetermined intervals, 100 μL of solution in various groups were withdrawn and quenched with 100 μL of acetonitrile containing 0.1% trifluoroacetic acid (TFA). HPLC analysis was performed on an LC-10AT VP Plus liquid chromatography system with an SPD-10 A VP Plus UV–Vis detector operating at 210 nm and quantified using Shimadzu LC solution Lite. The column used was a C18 reverse-phase column, and the mobile phase was composed of solvent A (water containing 0.1% TFA) and solvent B (70% acetonitrile and 0.1% TFA); the injection volume and flow rate were respectively set as 20 μL and 1.00 mL∙min^−1^. The appropriate calibration curve was first derived to calculate the concentration of thymopentin based on the above-mentioned procedure (Figure 5a). As shown in Figure 5b, upon exposure to rat plasma for 30 min, free thymopentin underwent rapid degradation and became undetectable, well matching that recorded in the literature. In sharp contrast, complexation by ExBP3C could maintain 24.85% of the original amount for 60 min. Additionally, two popular macrocycles, CP5A and CB[7], which could respectively recognize Lys, Arg, and Tyr, were chosen as control groups [26,37]. Under the same experimental conditions, co-dosing with either CP5A or CB[7] displayed no obvious benefit to the plasma stability of thymopentin. This finding was quite reasonable in that such traditional macrocycles had no effective protective potency for degradation sites at the peptide main chain via recognizing specific amino acid residues.

**Figure 6:**
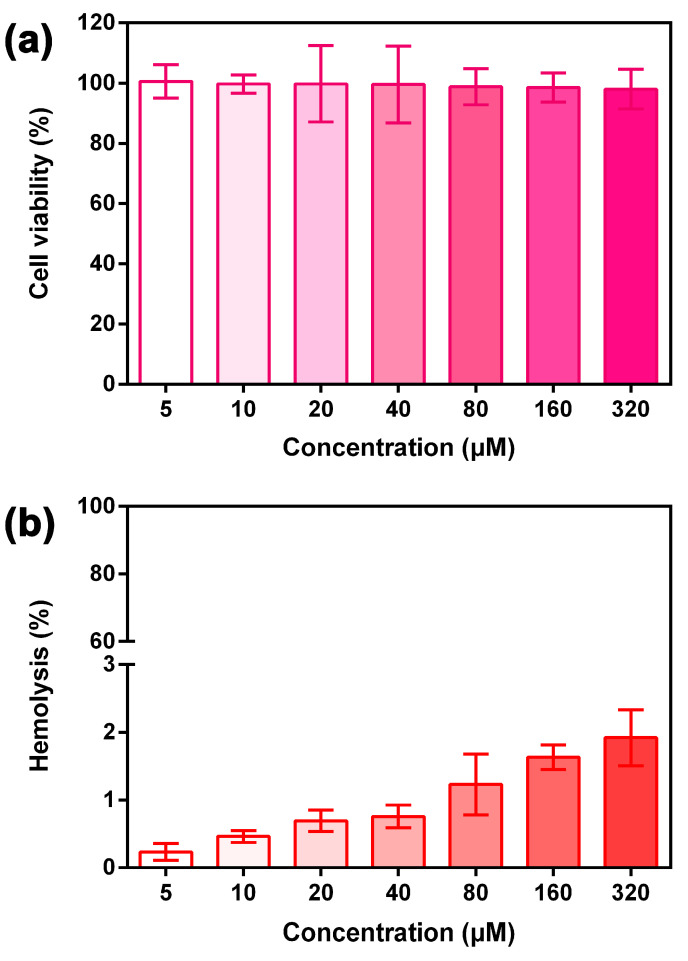
(**a**) Relative viability of mouse fibroblast cell line (L929) after treatment with ExBP3C for 24 h at the indicated concentrations (mean ± SD, *n* = 5). (**b**) Hemolysis of ExBP3C toward rabbit red blood cells (rRBCs) after 24 h of incubation time, and the hemolytic rate of rRBCs incubated with 1% Triton X-100 was defined as 100% (mean ± SD, *n* = 3).

In practical biomedical applications, the safety profiles of supplementary materials are of great concern. Here we preliminarily investigated the cytotoxicity of ExBP3C on a mouse fibroblast cell line (L929) and its hemolytic activity toward rabbit red blood cells (rRBCs). ExBP3C was first dissolved in deionized water and diluted to the required concentrations. For cytotoxicity studies, ExBP3C samples were added to L929 cell-containing wells, which were incubated at 37 °C under 5% CO_2_ for 24 h, and then the Cell Counting Kit-8 reagent was added into each well to stain for 30 min. The plate was measured at 450 nm using a plated reader, and the examination result indicated that ExBP3C possessed minimal cytotoxicity against L929 cells, even at relatively high concentrations (Figure 6a). For the hemolysis assay, ExBP3C samples were added to an erythrocyte suspension at 37 °C for 24 h. After that, the suspension was centrifuged at 1000 rpm for 10 min, and 100 μL of supernatant was transferred to a 96-well plate for measurement by a plate reader. As shown in Figure 6b, the hemolytic rate of rRBCs incubated with 1% Triton X-100 was defined as 100%, and the relative hemolysis of 320 μM of ExBP3C was determined to be only 1.92%. On the basis of experimental results and prior studies [34], we concluded that the administration of ExBP3C at the above examination range was relatively nontoxic on a cellular level.

## 3. Conclusions

In summary, a general supramolecular approach was proposed to improve the plasma resistance of peptides via entire encapsulation at its main chain. Taking large-sized ExBP3C as a macrocyclic host, a commercially available immunomodulator, thymopentin, could be recognized with a binding stoichiometry of 1:1 and a *K*_a_ value of 1.87 × 10^5^ M^−1^. Metabolic stability studies demonstrated that entire complexation by ExBP3C could significantly improve the stability of thymopentin in the presence of rat plasma. Control experiment results also revealed that traditional macrocycles, like CP5A and CB[7], were unable to provide effective protection to such peptide agents via recognition of its side chain. In vitro cytotoxicity and hemolysis studies revealed that the administration of ExBP3C was relatively safe on a cellular level within the examination range. This supramolecular approach to provide benefits without a structure change might be beneficial to other peptide agents. Studies directed at exploring this possibility are ongoing in our laboratory.

## Data Availability

The original contributions presented in this study are included in the article. Further inquiries can be directed to the corresponding author.
